# Optimizing High Pressure Processing Parameters to Produce Milkshakes Using Chokeberry Pomace

**DOI:** 10.3390/foods9040405

**Published:** 2020-04-01

**Authors:** Elena Diez-Sánchez, Antonio Martínez, Dolores Rodrigo, Amparo Quiles, Isabel Hernando

**Affiliations:** 1 Department of Food Technology, Universitat Politècnica de València, Camino de Vera s/n, 46022 València, Spain; eldiesan@upvnet.upv.es (E.D.-S.); mquichu@tal.upv.es (A.Q.); 2 Instituto de Agroquímica y Tecnología de Alimentos (IATA-CSIC), Paterna, 46980 València, Spain; amartinez@iata.csic.es (A.M.); lolesra@iata.csic.es (D.R.)

**Keywords:** antioxidant capacity, microbial inactivation, image analysis, high pressure processing, total phenolic content

## Abstract

High hydrostatic pressure is a non-thermal treatment of great interest because of its importance for producing food with additional or enhanced benefits above their nutritional value. In the present study, the effect of high hydrostatic pressure processing parameters (200–500 MPa; 1–10 min) is investigated through response surface methodology (RSM) to optimize the treatment conditions, maximizing the phenol content and antioxidant capacity while minimizing microbiological survival, in milkshakes prepared with chokeberry pomace (2.5–10%). The measurement of fluorescence intensity of the samples was used as an indicator of total phenolic content and antioxidant capacity. The results showed that the intensity of the treatments had different effects on the milkshakes. The RSM described that the greatest retention of phenolic compounds and antioxidant capacity with minimum microbiological survival were found at 500 MPa for 10 min and 10% (*w*/*v*) chokeberry pomace. Therefore, this study offers the opportunity to develop microbiologically safe novel dairy products of high nutritional quality.

## 1. Introduction

Nowadays, consumers show increasing preference for foods with additional or enhanced benefits beyond their basic nutritional value. These benefits come from the composition, e.g., bioactive compounds, which may have long-term health effects. There is convincing evidence of the cardioprotective effects for frequent consumption of fruits and vegetables high in fiber, micronutrients, and several phytochemicals. Specifically, the association between flavonoids and an increased cardiovascular health has been proven in berries [1]. Producing berry-based juice generates by-products, comprising peel and seeds, having a high nutritional value because of their polyphenol and fiber content. Berry by-products can be a value-added food ingredient [2,3,4] and recent studies show that the enrichment of food products with these by-products is feasible [5,6,7]. In this context, chokeberry (*Aronia melanocarpa*) pomace can be used, as chokeberry is one of the richest plant sources of phenolic phytochemicals, including procyanidins and anthocyanins [8,9] which are related to effectiveness in several pathological conditions where damage is caused by uncontrolled oxidative processes [10].

Previous studies have used dairy products and pomace for the production of yogurts with apple pomace [11,12,13] or fermented milk with grape pomace [14]. However, in the work of Issar et al. [11] and de Souza et al. [14], polyphenols and fiber were extracted, respectively, and added to milk for product preparation. Wang et al. [12,13] incorporated the apple pomace directly into the dairy matrix. Regarding berry pomace, Ni et al. [15] formulated yogurts with aqueous berry extracts from salal berry and blackcurrant pomace. In this study, we propose the incorporation of the pomace directly into the milk using high hydrostatic pressure (HPP) to help polyphenols being extracted into the matrix.

HPP is a method to preserve food and has the potential to retain or improve the bioaccessibility and bioavailability of nutritional and antioxidant compounds because of microstructural modifications [16]. HPP retains the nutritional and sensory quality of food products, but there is a concern related to food safety [17]. In this context, high pressures have been effective at inactivating vegetative cells when sufficient intense pressure is applied [18].

Thus, the present study aimed to prepare milkshakes enriched with polyphenols by adding chokeberry pomace to the milk and using HPP to improve polyphenols extraction from the pomace. The effect of high HPP parameters such as time and pressure on total phenolic content (TPC), antioxidant capacity (AC), and the microbiological inactivation in milkshakes with different concentrations of chokeberry pomace will be studied. To define the best processing conditions, response surface methodology (RSM) was used to maximize the TPC and the AC results while minimizing the microbiological survival.

## 2. Materials and Methods

### 2.1. Sample Preparation and HPP Treatments

Döhler GmbH (Darmstadt, Germany) provided fresh chokeberry pomace. The pomace was dried at 70 °C for 3 h and milled in a ZM 100 ultracentrifuge mill (Retsch GmbH, Haan, Germany) at 14,000 r.p.m. using a 0.5 mm sieve [19]. Reconstituted skimmed milk powder (Corporación Alimentaria Peñasanta S.A., Siero, Spain) was selected for chokeberry pomace inclusion.

Different concentrations of chokeberry powder were added to skimmed milk samples to give final chokeberry pomace concentrations of 2.5%, 6.25%, and 10% (*w*/*v*). The samples were poured into 50 mL polypropylene tubes that were introduced into polyethylene bags and heat-sealed (MULTIVAC Thermosealer, Switzerland) before undergoing HPP treatment. HPP treatments were performed in a unit with a 2.35 L vessel volume and maximum operating pressure of 600 MPa (High Pressure Food Processor, EPSI NV, Belgium). The samples were pressurized at 200, 350, and 500 MPa, at 18–22 °C, for 1, 5.5, and 10 min, using a compression rate of 300 MPa/min and a decompression time < 1 min, [20,21]. Other parameters, pressure intensity, pressurization time, and temperature were automatically controlled. Once the treatment was completed, the samples were taken from the vessel, immersed in an ice-water bath, and refrigerated (3 ± 1 °C) until use. Before each analysis, both microbiological and chemical, the samples were filtered with paper filter previously sterilized using an autoclave. The microbiological cultures and microscopic observations were immediately conducted after the filtration while the samples for the TPC and AC determination were stored by deep-freezing at −80 °C until use.

### 2.2. Total Phenolic Content

The total phenolic content (TPC) was determined according to the method described by Singleton et al. [22], with some modifications. The treated chokeberry pomace milk (5 mL) was homogenized in an Ultraturrax with 25 mL of 960 g kg^−1^ ethanol. The homogenate was centrifuged (4122 g, 30 min, 4 °C), filtered, and the supernatant was stored. This treatment was repeated on the leftover pellet using 25 mL of 960 g kg^−1^ ethanol to extract more supernatant, then added to the first supernatant; the total supernatant was brought up to 100 mL. After, 6 mL of distilled water and 0.5 mL of Folin-Ciocalteu reagent (1:1 (*v*/*v*)) were added to an aliquot of 1 mL of the ethanolic extract. After three minutes, 1 mL of sodium carbonate solution (20% (*w*/*v*)) (Panreac Química SLU, Castellar del Vallès, Barcelona, Spain) and 1.5 mL of distilled water were added. The mixture was vortexed and kept at room temperature (~25 °C) in a dark room for 90 min. Absorbance was measured at 765 nm using a spectrophotometer (series 1000, model CE 1021; CECIL Instruments Ltd., Cambridge, UK) with results expressed as mg of Gallic Acid Equivalents (GAE)/100 mL. Total phenolic extractions were made in triplicate.

### 2.3. Antioxidant Capacity

The antioxidant capacity (AC) was measured by a ferric reducing antioxidant power assay (FRAP) [23,24]. Extracts were obtained in the same way as for TPC determination. Distilled water (30 μL), the sample (30 μL), and FRAP reagent (900 μL) were placed in the cuvette. The cuvettes were incubated for 30 min in a water bath at 37 °C and the absorbance was measured at 595 nm. A calibration curve was obtained using different concentrations of Trolox in 960 g kg^−1^ ethanol. The results were expressed as μmol Trolox/mL of sample. Three separate extractions were made for each treatment and the measurements were performed in triplicate.

### 2.4. Chokeberry Microstructure

The microstructure analysis was carried out following Hernández-Carrión et al. [25] with some modifications. For the study of the chokeberry microstructure, a Nikon Eclipse E80i microscope (Nikon, Tokyo, Japan) was used. The autofluorescence of the phenolic compounds in the samples was observed using a mercury arc lamp with a tetramethyl rhodamine filter (λ_ex_ = 543/22 nm, λ_em_ = 593/40 nm) as the excitation source. Samples were visualized using ×10 and ×20 objective lenses. The micrographs were stored at a 1280 × 1024-pixel resolution using the microscope software (NIS-Elements F, Version 4.0, Nikon, Tokyo, Japan). Analysis of the fluorescence intensity was conducted with the ImageJ software.

### 2.5. Microbiological Analysis

The effect of HPP treatment was evaluated on natural contaminating flora (aerobic mesophilic microorganisms, molds, and yeasts) and on *Listeria monocytogenes* serovar 4b [26] (CECT 4032) as pathogen microorganism [27]. The growth media used for the spreading of samples was plate count agar (Scharlau Chemie S. A., Sentmenat, Spain) for mesophilic aerobic; potato dextrose agar (Scharlau Chemie S. A., Sentmenat, Spain) for molds and yeasts; and tryptic soy agar (Scharlau Chemie S. A., Sentmenat, Spain) for *L. monocytogenes*. The incubation conditions were 48 h at 30 °C, 120 h (5 days) at 24 °C, and 48 h at 37 °C, respectively.

*L. monocytogenes* was artificially inoculated in the sample. The stock vials containing *L. monocytogenes* at a concentration ca. 10^9^ cfu/mL were generated following the methods described by Saucedo-Reyes et al. and Pina-Pérez et al. [20,28]. Before HPP treatment, vials were inoculated in the chokeberry-skimmed milk samples at a final concentration of 10^8^ cfu/mL. The counts for evaluating microorganism inactivation were performed before and after each HPP treatment. Two aliquots (0.1 mL) were taken from each sample, diluted with buffered peptone water (Scharlau Chemie S. A, Sentmenat, Spain), and plated in the respective agar. Two replicas of each treatment were obtained, and three repetitions of each treatment condition was performed. The survival fraction S = N/N_0_ and the level of inactivation Log_10_ (N/N_0_) were evaluated for each repetition.

### 2.6. Experimental Design and Statistical Analysis

RSM was used to optimize the preservation process and to investigate the simultaneous effects of pressure, time, and chokeberry powder concentration on TPC, AC, and microbiological inactivation of the prepared samples. For the chokeberry-milk matrix, a face-centered central composite design was used with three levels (maximum, minimum, and central) and three independent factors, namely pressure (200 to 500 MPa), time (1 to 10 min), and chokeberry pomace concentration (2.5 to 10% (*w*/*v*)), resulting in 16 combinations (Table 1). The central point of the three variables was replicated twice to assure the reproducibility and stability of the results. All the experiments were randomized. A quadratic model was obtained with regression coefficients associated with the linear, quadratic, and interaction effects. A *t*-test determined their significance through the *p*-value generated.

The non-significant terms (*p* > 0.05) were deleted from the second-order polynomial model after an ANOVA test and a new ANOVA was performed to find the coefficients of the final equation for better accuracy [29]. The experimental design and the data analysis were performed using the Statgraphics^®^ Centurion XVII software (Statpoint Technologies, Inc., Warrenton, VA, USA).

## 3. Results and Discussion

### 3.1. Effect of HPP on TPC, AC, and Microbial Counts of Chokeberry Milkshakes

Effects of HPP treatments on the TPC, AC, and microbiological survival fraction are shown in Table 2. The analyses were conducted on untreated and treated samples to observe differences with HPP treatment. Results for molds and yeasts are not shown because there were no changes in any treatments.

TPC concentration in untreated samples with 2.5%, 6.25%, and 10% (*w*/*v*) of chokeberry pomace is 53.02 ± 0.14, 73.92 ± 3.17, and 121.16 ± 0.31 mg GAE/100 mL, respectively. Furthermore, the AC results for samples 2.5, 6.25, and 10% (*w*/*v*) of chokeberry pomace, are 6.06 ± 0.14, 9.27 ± 0.20, and 14.89 ± 0.30 µmol Trolox/mL, respectively. As expected, the TPC and AC results are higher with higher pomace concentrations.

In treated samples, the highest result for TPC is 155.28 ± 2.07 mg GAE/100 mL with 500 MPa during 1 min and 10% (*w*/*v*) pomace addition; the lowest is 42.45 ± 2.89 mg GAE/100 mL with 350 MPa during 5.5 min and 2.5% (*w*/*v*) pomace addition. Yet, the highest AC is 17.3 ± 1.08 µmol Trolox/mL for the treatment of 200 MPa during 1 min with 10% (*w*/*v*) pomace addition. The treatment that obtained the lower AC matches the one that obtained the lower TPC (350 MPa during 5.5 min with 2.5% (*w*/*v*) pomace addition).

The lowest results for TPC and AC are obtained for the intermediate pressure and not for the lowest as expected, yet these low results are similar to other treatments at different processing conditions but with the same pomace concentration (2.5%). In contrast, the higher value results for TPC and AC are obtained for milkshakes with 10% of chokeberry. When comparing the results of the treatments at each concentration, values were higher as the pomace concentration increased. In addition, comparing the results of treatments at each concentration with its untreated counterpart, samples 6.25 and 10% show an increase in TPC and AC, influenced by the pressures and times studied. However, for samples at 2.5%, this effect is less acute, affected only by high pressures (500 MPa).

Thus, the results are affected by all the factors in this study (pressure, time, and concentration), but mainly the pomace concentration. The HPP treated foods are either unaffected or have increased TPC and/or extractability following treatments with high pressures [30]. Andrés et al. [31] found increases of 6.6% and 4.2% in TPC values for fruit smoothies treated at 450 and 600 MPa, respectively. Corrales et al. [32] showed that treating at 600 MPa enhanced the anthocyanin extraction and its AC in grape by-products than with conventional extraction methods. Liu et al. [33] found treatments at 200 MPa, for 5 and 10 min, led to an increased TPC of 14.24% and 14.35% in wild *Lonicera caerulea* berry, respectively, however, for treatments at 500 and 600 MPa there was a significant decrease of TPC. In contrast, other authors found HPP had little effect on phenolic content. Barba et al. [34] observed TPC to be relatively resistant to the effect of processing in tomato purées. Hurtado et al. [35] did not observe differences in AC values between untreated and treated red fruit-based smoothies for treatments at 350 MPa, 10 °C, and 5 min. Patras et al. [36] found that phenol content in HPP treated strawberry purées was relatively resistant to the effect of processing at 400 and 500 MPa, only showing an increase in treatments at 600 MPa for TPC and AC. Therefore, the results obtained with HPP depend of several conditions, such as the matrix in which they are applied, and the severity of the treatment and it is necessary to study the behavior of different samples with these treatments.

**Figure 1:**
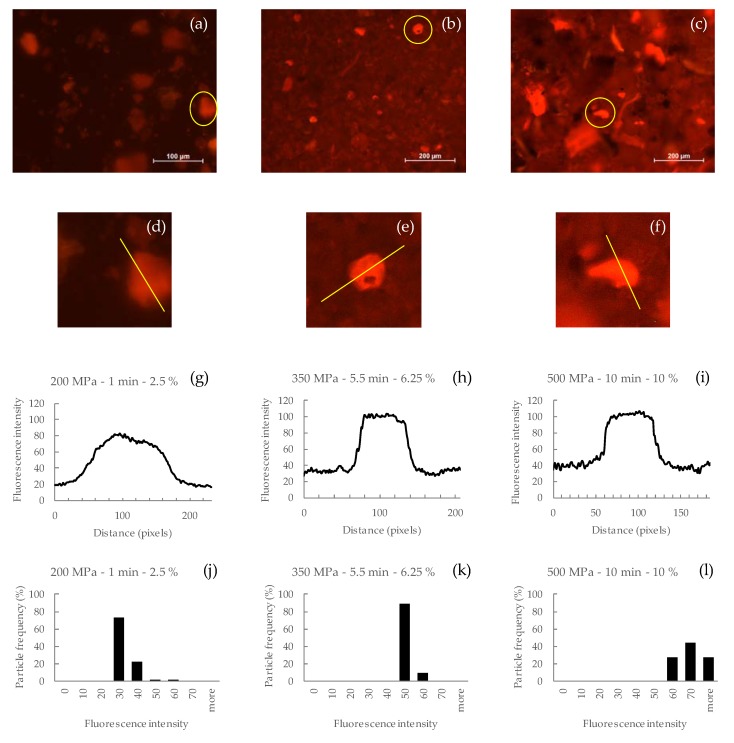
Fluorescence intensity of: 200 MPa, 1 min, 2.5% (**a**,**d**,**g**,**j**); 350 MPa, 5.5 min, 6.25% (**b**,**e**,**h**,**k**); and 500 MPa, 10 min, 10% (**c**,**f**,**i**,**l**).

In fluorescence microscopy, the intensity value of a pixel is related to the number of fluorophores present at the corresponding area in the particle. Thus, the digital images can be used to extract the intensity values to determine the local concentration of fluorophores in a specimen [37]. In our case of study, the images in Figure 1 show the pomace particles dispersed into the milk matrix.

To analyze the florescence intensity the images corresponding to the lower (200 MPa, 1 min, 2.5%), central (350 MPa, 5.5 min, 6.25%), and higher (500 MPa, 10 min, 10%) treatments were selected. The particle with greater intensity was selected to generate intensity profiles (Figure 1a–c). A line (shown in yellow) was drawn across the particle, and a plot (graph) was generated to show the intensity values of the pixels along the line (Figure 1d–i). In addition, Figure 1j–l shows the relation between the percentages of particles at each fluorescence intensity interval.

The fluorescence intensity for the isolated particles is higher in the medium (Figure 1k) and high treatments (Figure 1l) than in the low treatment (Figure 1j). Comparing the background intensity, corresponding to the liquid phase of the sample, fluorescence increases as the severity of the treatment increases. Several authors [16,38,39] have described the cell wall degradation and breakage in plant tissue after HPP, leading to a leaching of contents from the pomace cells (included polyphenols) to the milk acting as a liquid medium [32,38]. In addition, Gonzalez and Barrett [40] described that treatments at pressures above 220 MPa were responsible for the breakage of the membrane structure because of protein unfolding and interface separation. Therefore, as higher pressures are applied, the phenolic contents are being released to the medium due to the membrane breakage, giving as a result higher values of fluorescence intensity. The particle frequency plots show that the frequency of particles at high intensities rises with the severity of the treatment. These results agree with the results of TPC and AC, i.e., higher fluorescence intensities correspond to the treatments that obtained the higher TPC and AC results. Therefore, measurement of fluorescence intensity can be an indicator for TPC and AC in this type of sample. Further research is needed to prove if the analysis is usable in other matrices.

Besides the effect of HPP on the polyphenols, there could be a microbial inactivation because of changes induced in the microbial cells. These changes include alterations in the cell membrane, effects on proteins, and effects on the genetic mechanism of microorganisms [18,41,42]. Seen in microbiological inactivation results in Table 2, treatments at 200 MPa during 1 min with 2.5% (*w*/*v*) of pomace and at 200 MPa during 10 min with 2.5% of pomace do not show microbial inactivation. At 2.5% pomace concentration and low pressure (200 MPa), longer treatment time is not enough for microbial inactivation. Muñoz-Cuevas et al. [43] also observed this behavior. Still, it is necessary to reach a minimum treatment intensity (500 MPa, 10 min) to obtain significant *L. monocytogenes* inactivation. At this condition, an increase in chokeberry pomace concentration from 2.5% to 10% (*w*/*v*) increases microbial inactivation from 3.63 to 4.02 Log reductions.

Thus, increasing the pressure and treatment time results in an increase in the lethal effect of HPP treatment. This effect relates to food composition, technological parameters, and the factors acting in synergy [44,45].

**Figure 2:**
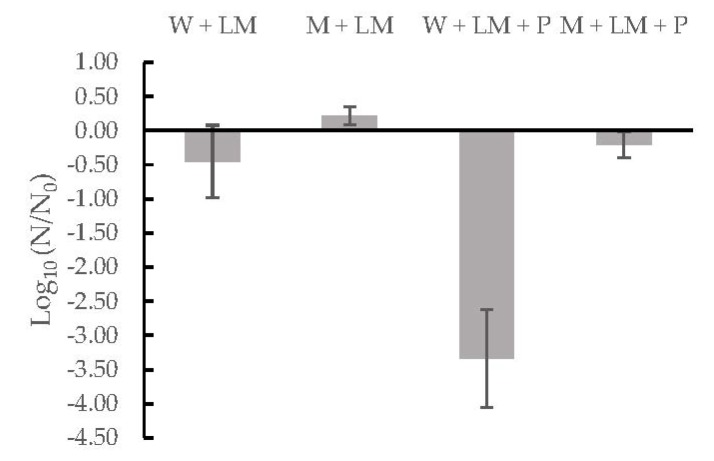
Inactivation level of ingredients’ different combinations: peptone water (W), milk (M), inoculated *Listeria monocytogenes* (LM), and pomace (P).

Besides the effect of treatment conditions, several authors have described the high antimicrobial capacity of berry fruits and their by-products [46,47,48] and the synergistic effect between natural substances and high pressure treatments [28,49,50]. Despite the evidence found in the literature, except the treatments of 500 MPa, 10 min with 2.5% and 10% (*w*/*v*) pomace, the inactivation values are lower than similar treatments with other products and microorganisms. For example, Evrendilek & Balasubramaniam [49] concluded that samples of ayran (yogurt drink) treated at 600 MPa during 5 min had reduced in the levels of *L. monocytogenes* and *L. innocua* by more than five log units (*p* < 0.05) at ambient temperature. Nevertheless, Gervilla et al. [51] and Black et al. [52] have described a baroprotective effect that milk has on the cells. Thus, this effect could counteract the antimicrobial effect of chokeberry pomace, explaining the low inactivation levels found for *L. monocytogenes* in this study. To prove this effect, an experiment was conducted where the central point of the design (350 MPa, 5.5 min in milk with 6.25% (*w*/*v*) of chokeberry pomace) was used as a treatment to compare the inactivation reached in four different matrices: (i) peptone water with *L. monocytogenes*, (ii) milk with *L. monocytogenes*, (iii) peptone water with *L. monocytogenes* and chokeberry pomace, and (iv) milk with *L. monocytogenes* and chokeberry pomace. Results tested the baroprotective effect of milk and are shown in Figure 2.

In samples without milk (W + LM and W + LM + P), the number of surviving cells is lower than with milk samples (M + LM and M + LM + P), and more prominent when pomace is added. Apart from the protective effect of milk, an increase is seen in the efficacy of HPP against *L. monocytogenes* when pomace is present in the sample. Thus, these results can describe the synergistic effect of pomace, HPP, and the protective effect of milk on *L. monocytogenes*. Still, there is microbial inactivation with some treatments, even with the protective effect of milk on the microbiological cells.

### 3.2. Processing Parameter Optimization and Their Effect on the Safety and Quality of the Formulated Matrix

The best processing conditions for treating chokeberry milkshakes when HPP is combined with added by-products with antimicrobial and antioxidant properties (chokeberry pomace) were studied by RSM. This methodology uses a sequence of designed experiments to obtain an optimal response.

**Figure 3:**
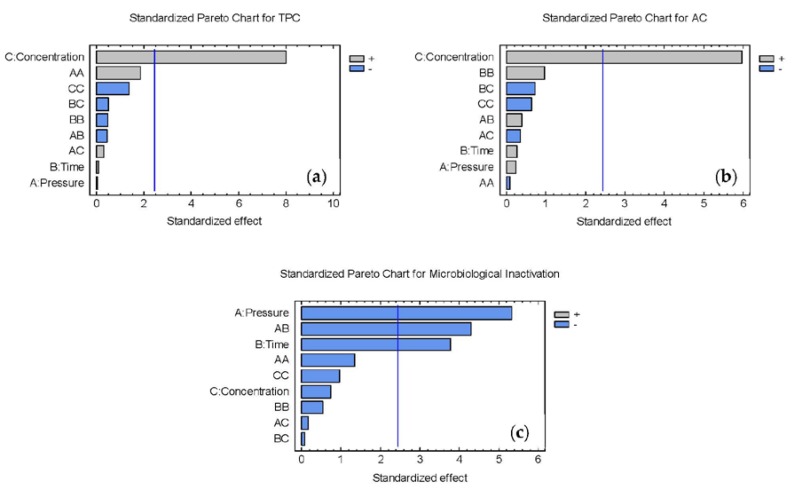
Estimated effects of each factor (pressure, time, and concentration) and their interactions with TPC (**a**), AC (**b**), and *L. monocytogenes* inactivation (**c**) where + or − means a positive or negative relation between factor (pressure, time or concentration) and response (TPC, AC or L. monocytogenes survival fraction), respectively. A: pressure, B: time and C: concentration. The combination of letters (AA, BB, AB …) refer to the interactions carried out in the analysis.

First, the estimated effects of each factor (pressure, time, and concentration) and their interactions were analyzed (Figure 3). The response function for the factors and the adjusted regression coefficient (*R*^2^ adjusted), showing the percentage of variation in the response explained by the fitted model, is shown in Equations (1)–(3) (pressure (P), time (t), chokeberry pomace concentration (C)). The value of the adjusted *R*^2^ close to one indicates a high correlation between the experimental and fitted values.

(1) $$\mathrm{TPC}=25.6475+11.2687\times C\;R^{2}\mathrm{adj}=0.85$$

(2) $$\mathrm{AC}=2.08477+1.38395\times C\;R^{2}\mathrm{adj}=0.80$$

(3) $${\mathrm{Log}}_{10}\left(\frac{N}{N_{0}}\right)=-0.160277+0.000618685\times P+0.285165\times t-0.00123019\times P\times t\;R^{2}\mathrm{adj}=0.76$$

Figure 3 shows the pareto chart for TPC, AC, and microbial inactivation. This chart determines the magnitude and the importance of the effects. The bars that extend beyond the line correspond to effects that are statistically significant with a 95.0% confidence level. The factor “pomace concentration in milkshakes” is the only factor that significantly affects TPC and AC concentration. However, the results in Table 2 show TPC and AC are influenced by all the factors, including time and pressure. Chokeberry pomace has been reported as a berry fruit with high phenolic content [8]. Thus, though it could exist with the effect of pressure and time, the results could be masked by the natural high phenolic content.

The low effect of treatment conditions on TPC and AC could be explained by using milk as a liquid medium. High pressure processing induces physicochemical and technological changes in milk properties. When HPP is applied to milk, the casein micelles are disintegrated into casein particles of smaller size, which is accompanied by an increase in casein and calcium phosphate levels in the serum phase of milk and by a decrease in both non-casein nitrogen and serum nitrogen fractions [18,53]. In addition, interactions between polyphenols and milk proteins have been previously described by other researchers [54,55,56]. In our work, these interactions could be favored by the changes in the casein structure due to HPP treatment, which would lead to the formation of complexes that restrict the accessibility of analysis, leading to lower AC and TPC and a non-significant effect of treatment conditions (pressures and time). Tadapaneni et al. [57] also observed this effect in strawberry-based beverages treated with HPP at pressures ranging from 200 to 600 MPa. They saw, when formulated with milk instead of water, the beverage presented reduced levels of AC and anthocyanins because of complexes forming between polyphenols and milk proteins. Therefore, as the effect of concentration is so pronounced in RSM and polyphenol–milk protein interactions may exist, decreasing the AC and TPC, the effect of the other parameters is much lower, leading to a non-significant effect of time and pressure.

In contrast, pressure and time are the parameters with a significant effect on the microbiological inactivation. Thus, the chokeberry pomace concentration added to the milkshake, does not have a significant effect on the inactivation results. These results confirm the hypothesis explained above (Figure 2); there is an antimicrobial effect of berry pomace. However, it is masked with the protective effect of milk on *L. monocytogenes* cells, giving as a lower inactivation result than with similar treatment conditions in products with a natural antimicrobial agents, yet without milk [49]. Despite the protective effect of milk on microorganisms, adding chokeberry pomace could help achieve higher inactivation levels than HPP without the pomace.

Once the estimated effect and its interaction were analyzed, the response optimization was carried out. The results show that the optimized factors are 500 MPa for 10 min in milk with 10% (*w*/*v*) of chokeberry pomace (Table 3). This treatment condition ensures the maximum TPC and AC with the minimum microbiological survival.

The optimum treatment conditions are the same as those of the experimental design. When comparing the results in Table 2 with the predicted values in Table 3, we see that experimental results are like the predicted values through optimization. Therefore, the RSM is proven to be a reliable tool to predict the behavior of the sample studied in terms of AC, TPC, and microbial inactivation.

## 4. Conclusions

The fluorescence intensity measurement of microstructure images can be an indicator of the TPC and AC of the samples. Microstructure images showed that, with intense treatment, there is leaching of the polyphenolic substances into the milk because of cell structure breakage. For the microbiological inactivation, the results showed that the pomace had antimicrobial properties, but they were partially masked by the interactions between milk proteins and the polyphenols available, and that higher levels of inactivation were achieved at high pressures and long treatment times. The RSM results showed that TPC and AC were only affected by the pomace concentration added to the milkshake, because the high pomace concentration and the polyphenol–milk protein interactions could mask the effect of pressure and time.

Although the efficiency of HPP inactivation on *L. monocytogenes* has been proven, further research is needed for products without milk to study the effect of chokeberry pomace treated with HPP on the TPC, AC, and microbial inactivation.

## Figures and Tables

**Table 1:** Experimental design matrix for studies conducted.

Run	Pressure (MPa) (X_1_)	Time (min) (X_2_)	Chokeberry Pomace (% (*w*/*v*))
1	500	1	2.5
2	350	10	6.25
3	500	5.5	6.25
4 ^a^	350	5.5	6.25
5	500	10	10
6	350	5.5	2.5
7	350	5.5	10
8	350	1	6.25
9	200	10	10
10	500	10	2.5
11	200	5.5	6.25
12	200	10	2.5
13	350	5.5	6.25
14	200	1	10
15	200	1	2.5
16	500	1	10

^a^ Central point.

**Table 2:** Effect of HPP and chokeberry pomace on TPC, AC, and the microbial survival fraction.

Pressure (MPa)	Time (min)	Chokeberry Pomace % (*w*/*v*)	TPC (mg GAE/100 mL)	AC (µmol Trolox/mL)	Inactivation Log_10_ (N/N_0_)
0	0	2.5	53.02 ± 0.14	6.06 ± 0.14	-
		6.25	73.92 ± 3.17	9.27 ± 0.20	-
		10	121.16 ± 0.31	14.89 ± 0.30	-
200	1	2.5	49.39 ± 2.24	5.02 ± 0.34	0.01 ± 0.08
		10	130.20 ± 3.46	17.3 ± 1.08	−0.18 ± 0.04
	5.5	6.25	136.79 ± 8.06	11.79 ± 0.99	−0.20 ± 0.08
	10	2.5	50.54 ± 4.64	4.80 ± 0.16	0.06 ± 0.12
		10	132.85 ± 2.31	13.98 ± 0.49	−0.11 ± 0.06
350	1	6.25	84.32 ± 2.59	10.13 ± 0.79	−0.20 ± 0.06
	5.5	2.5	42.45 ± 2.89	4.77 ± 0.39	−0.25 ± 0.14
		6.25 ^a^	78.54 ± 3.39 ^a^	7.58 ± 0.41 ^a^	−0.21 ± 0.19 ^a^
		6.25	106.14 ± 4.28	8.54 ± 0.03	−0.33 ± 0.09
		10	124.96 ± 3.78	16.45 ± 0.10	−0.55 ± 0.05
	10	6.25	101.92 ± 5.70	16.53 ± 0.78	−0.33 ± 0.32
500	1	2.5	54.14 ± 0.61	5.7 ± 0.45	−0.44 ± 0.05
		10	155.28 ± 2.07	16.36 ± 0.35	−0.67 ± 0.08
	5.5	6.25	97.17 ± 7.09	11.33 ± 2.03	−0.87 ± 0.06
	10	2.5	58.36 ± 2.97	6.69 ± 0.19	−3.63 ± 0.08
		10	134.17 ± 1.57	14.79 ± 0.76	−4.02 ± 0.15

^a^ Central point; HPP: High Pressure Processing; TPC: Total Phenolic Content; AC: Antioxidant Capacity; N: final cell concentration; N_0_: initial cell concentration.

**Table 3:** Predicted and limit response values for optimum treatment conditions.

Response	Predicted	Lower 95.0% Limit	Upper 95.0% Limit
TPC (mg GAE/100 mL)	138.33	125.68	150.99
AC (µmol Trolox/mL)	15.92	14.13	17.72
Log_10_ (N/N_0_)	−3.15	−3.96	−2.34
